# Immersive virtual reality assessments of working memory and psychomotor skills: A comparison between immersive and non‐immersive assessments

**DOI:** 10.1111/jnp.70014

**Published:** 2025-10-07

**Authors:** Panagiotis Kourtesis, Andrea Lizarraga, Sarah E. MacPherson

**Affiliations:** ^1^ Department of Psychology The University of Edinburgh Edinburgh UK; ^2^ Department of Psychology The American College of Greece Athens Greece; ^3^ Department of Informatics & Telecommunications National and Kapodistrian University of Athens Athens Greece; ^4^ Department of Psychology National and Kapodistrian University of Athens Athens Greece

**Keywords:** digital literacy, eye‐tracking, neuropsychological tests, psychomotor skills, usability, virtual reality, working memory

## Abstract

**Objective:**

Immersive virtual reality (VR) enhances ecological validity and facilitates intuitive and ergonomic hand interactions for performing neuropsychological assessments. However, its comparability to traditional computerized methods remains unclear. This study investigates the convergent validity, user experience and usability of VR‐based versus PC‐based assessments of short‐term and working memory, as well as psychomotor skills, while also examining how demographic and IT‐related skills influence performance in both modalities.

**Methods:**

Sixty‐six participants performed the Digit Span Task (DST), Corsi Block Task (CBT) and Deary‐Liewald Reaction Time Task (DLRTT) in both VR‐ and PC‐based formats. Participants' experience in using computers and smartphones, and playing videogames, was considered. User experience and system usability of the formats were also evaluated.

**Results:**

While performance on DST was similar across modalities, PC assessments enabled better performance on CBT and faster reaction times in DLRTT. Significant correlations between VR and PC versions supported convergent validity. Regression analyses revealed that performance on PC versions was influenced by computing and gaming experience, whereas performance on VR versions was largely independent of these factors, except for gaming experience predicting performance on CBT backward recall. Moreover, VR assessments received higher ratings for user experience and usability than PC‐based assessments.

**Conclusion:**

Immersive VR assessments provide an engaging alternative to traditional computerized methods, with minimal reliance on prior IT experience and demographic factors. This resilience to individual differences suggests that VR may offer a more equitable and accessible platform for automated cognitive assessment. Future research should explore the long‐term reliability of VR‐based assessments.

## INTRODUCTION

Cognitive abilities underpin a wide spectrum of human behaviour, ranging from intricate problem‐solving and learning tasks to routine, everyday activities. Within this domain, working memory and psychomotor skills have attracted extensive interest in research and clinical contexts due to their profound impact on functional independence and overall quality of life (Alloway et al., 2009; Baddeley, 1992; Deary & Der, 2005a). Broadly, working memory enables the temporary storage and manipulation of information, forming the backbone of higher‐level processes such as reasoning, language comprehension and coherent response formation (Gathercole et al., 2008; Raghubar et al., 2010; Rogers et al., 2011). Psychomotor skills, by contrast, integrate sensory input, cognitive appraisal and motor output, allowing humans to navigate their environment effectively, such as through basic object manipulation or more complex tasks like driving or operating machinery (Chaiken et al., 2000). Both working memory and psychomotor abilities are susceptible to age‐related decline and to neuropathological conditions, such as stroke or dementia, reinforcing the need for robust, reliable assessment tools (Alloway et al., 2009; Hatem et al., 2016).

The neuropsychological evaluations of these constructs have relied on well‐established assessment tools such as the Digit Span Task (DST; Ramsay & Reynolds, 1995; Woods, Kishiyama, et al., 2011), Corsi Block Task (CBT; Corsi, 1973; Kessels et al., 2000) and reaction‐time measures like the Deary–Liewald Reaction Time Task (DLRTT; Deary et al., 2011). These assessments offer diagnostic clarity and standardized administration protocols, which are particularly advantageous for identifying cognitive impairment associated with neurological conditions such as Alzheimer's disease and stroke (Alloway et al., 2009; Petersen, 2004). The DST evaluates verbal short‐term or working memory by requiring individuals to repeat or manipulate sequences of digits (Baddeley, 1992), whereas the CBT assesses visuospatial short‐term or working memory by calling for participants to replicate block sequences in a forward or backward order (Corsi, 1973). Reaction‐time tasks, whether simple (SRT) or choice‐based (CRT), examine psychomotor coordination and they have been linked to fluid intelligence, everyday motor functioning, and even mortality risk (Deary & Der, 2005b; Der & Deary, 2006; Jensen, 2006).

Computerized versions of the DST, CBT and DLRTT have been suggested for automating administration and scoring in an attempt to mitigate human‐related bias in the neuropsychological evaluation of the targeted cognitive constructs (Deary et al., 2011; Kessels et al., 2000; Woods, Herron, et al., 2011). Despite their proven utility, these computerized versions face significant limitations in capturing the complexity of real‐world cognition. They predominantly rely on two‐dimensional, highly controlled stimuli and environments that diverge substantially from the multifaceted, three‐dimensional challenges people encounter in daily life (Kourtesis & MacPherson, 2021; Parsons, 2015). For instance, tasks like repeating digit sequences or selecting squares on a screen do not reflect the distractor‐loaded contexts and/or spatial characteristics of everyday scenarios. Consequently, these tests may have limited ecological validity, where they have reduced capacity to predict real‐life performance (veridicality) and/or emulate daily experiences (verisimilitude; Spooner & Pachana, 2006). While their simplicity and standardization help isolate specific cognitive processes, these instruments cannot replicate the embodied performance of tasks in a three‐dimensional, 360‐degree, real‐world setting, thus overlooking important visuospatial and motor aspects of everyday functioning.

An additional concern relates to digital literacy confounds associated with computerized assessment. A growing body of evidence suggests that people with higher familiarity in video gaming, computing or smartphone use often outperform less tech‐savvy participants on computerized tasks (Bauer et al., 2012; Feldstein et al., 1999; Iverson et al., 2009). Technology‐proficient individuals benefit from enhanced fine motor control, faster reaction times and more adept navigation through on‐screen interfaces (Borecki et al., 2013). Consequently, traditional computerized assessments requiring fine motor skills to press keys and/or control a mouse may inadvertently also measure digital proficiency rather than purely cognitive constructs. While this limitation applies to the general population, such a confound is particularly problematic when testing older adults or individuals from lower socioeconomic or educational backgrounds, who may lack consistent exposure to computers or gaming devices (Bauer et al., 2012; Feldstein et al., 1999). Thus, while computerized tasks facilitate automated data collection and scoring, they can introduce biases that undermine test fairness and inclusivity.

Immersive virtual reality (VR) emerges as a promising solution to these challenges, enabling researchers and clinicians to preserve the structured control of laboratory settings while introducing greater ecological validity. By using head‐mounted displays, motion tracking and optional haptic feedback, VR can simulate complex, three‐dimensional environments where participants interact via naturalistic and intuitive embodied interactions instead of keyboards or mice (Kourtesis et al., 2020). This shift may not only lessen the advantage conferred by prior computer experience but also offer a more intuitive means of completing cognitive tasks, as well as potentially improving usability, user experience and data fidelity (Kourtesis & MacPherson, 2021; Zaidi et al., 2018). Immersive VR neuropsychological assessments, such as the Virtual Reality Everyday Assessment Lab (Kourtesis et al., 2021) and Nesplora Aquarium (Climent et al., 2021), have demonstrated robust validity. Notably, such immersive formats appear to reduce test fatigue, sustain participant attention and accurately measure cognitive processes ranging from working memory to executive functions (Kourtesis et al., 2020).

Another strength of VR‐based methods lies in their capacity to systematically manipulate environmental factors. For example, clinicians can introduce or remove distractors, vary object placement and alter time pressures in ways that are challenging to replicate in physical or 2D computerized settings (Kourtesis et al., 2020; Parsons, 2015). As VR technology evolves, advanced features like eye‐tracking, motion sensors and multisensory integration (e.g., auditory or haptic cues) promise to reveal even more sophisticated insights into the cognitive and motor abilities involved in task performance (Kim et al., 2020; Kourtesis et al., 2020; Mäkinen et al., 2022). This level of detail could help pinpoint whether performance delays stem from slowed visual processing, decreased attention or impaired motor execution, thereby informing more targeted interventions.

Considering these potential advantages, we compared immersive‐VR and conventional PC (i.e., desktop/laptop personal computer formats presented on a 2D monitor, irrespective of the operating system) versions of three established neuropsychological tasks. The DLRTT and CBT were our primary targets, as their psychomotor speed and visuospatial‐WM demands should benefit most from embodied, depth‐rich interaction. By contrast, the DST was included as a verbal working memory control task, since its auditory, sequential nature and simple responses require minimal technological competency. Thus, we expected negligible VR–PC differences for DST, but more pronounced ones for the visuospatial and psychomotor tasks. Across all three tasks, we assess convergent validity, absolute performance, user experience and usability. Our principal hypothesis is that self‐reported gaming or computing experience will explain less variance in the VR formats, thereby testing whether immersive VR really mitigates technology‐related performance bias.

To achieve these aims, the study focuses on young adults. This group shows peak levels of cognitive and psychomotor functioning (Deary & Der, 2005a; Der & Deary, 2006), which helps control for age‐related variability and allows for a clearer evaluation of task performance across formats. Young adults also differ widely in their experience with digital technologies and gaming (Feldstein et al., 1999; Iverson et al., 2009; Zioga, Ferentinos, et al., 2024), making them well suited for testing whether immersive VR reduces performance biases linked to IT proficiency. Establishing the validity and usability of VR assessments in this population provides a necessary foundation before extending them to older or clinical groups (Kourtesis et al., 2020; Tuena et al., 2020).

## METHODS

### Participants

Sixty‐six participants (38 women) aged 18–45 years (*M* = 27.89, SD = 4.88), with 12–25 years of education (*M* = 16.65, SD = 2.70), were recruited through posters, email lists, social media and participant pools from the Psychology Departments of the American College of Greece and the University of Edinburgh. A priori power analysis (*f* ^2^ = .15, *α* = .05, 1 added predictor in each iteration and up to a 4‐predictor model) indicated that *N* = 55 would provide ≥80% power to detect a medium incremental effect. Our final sample of 66 therefore exceeded this requirement. Ethical approval was obtained from the PPLS Research Ethics Committee of the University of Edinburgh (318‐2223/8), ensuring accordance with the Helsinki Declaration. All participants provided written informed consent. Each participant received £15 (or the €18 equivalent) as compensation for their time.

### Materials

#### Hardware and software

VR tasks were administered using an HTC Vive Pro Eye headset with built‐in eye tracking. This system exceeds minimum recommended hardware specifications for reducing cybersickness (Kourtesis et al., 2019). VR software was developed in Unity 2019.3.f1 (Unity Technologies, 2020), following ergonomic guidelines (International Organization for Standardization, 2007) and best practices for VR in neuropsychology (Kourtesis et al., 2020). Interactions were managed via the SteamVR SDK, allowing participants to use hand controllers for naturalistic interaction. A Windows 11 Pro laptop with an Intel Core i9 CPU, 128 GB RAM, and a GeForce GTX 1060 Ti graphics card was used. Audio prompts were generated with Amazon Polly, and spatial audio was facilitated by the SteamAudio plugin. Computerized tasks were hosted on the PsyToolkit platform (Stoet, 2010, 2017).

#### Questionnaires

##### Demographic and IT Skills Questionnaire

A survey gathered basic demographic information, including age (in years), education (in years of formal schooling) and sex (male/female), and assessed computing and smartphone application experience both using two 6‐point Likert‐scale items (one on usage frequency, 1 = *Neve*r; 6 = *Every day*, and one on perceived ability, 1 = *No Skill*; 6 = *Expert*). Each domain's frequency and ability items were summed to produce a single experience score.

##### Gaming Skill Questionnaire

The Gaming Skill Questionnaire (GSQ, Zioga, Ferentinos, et al., 2024; Zioga, Nega, et al., 2024) was employed to assess participants' expertise across six gaming genres (i.e., sports games, first‐person shooter games, role‐playing games, action–adventure games, strategy games and puzzle games) by asking two 6‐point Likert‐scale items per genre: one on frequency of play (1 = *Never*, 6 = *Everyday*) and one on self‐perceived expertise (1 = *No Skill*; 6 = *Expert*). Each genre‐specific score consisted of the sum of these two items, and a total gaming skill score was calculated by summing across all six genres.

##### User Experience Questionnaire

A shortened, 8‐item version of the User Experience Questionnaire (UEQ) (Schrepp et al., 2017) was used to evaluate subjective impressions of task interfaces (VR vs. PC). Participants rated paired adjectives (e.g., ‘supportive–obstructive’) on a 7‐point Likert scale. Summing all items yielded a single user experience score with a maximum score of 56.

##### System Usability Scale

The System Usability Scale (Brooke, 1995, 2013) is a widely used 10‐item questionnaire measuring perceived usability on a 5‐point Likert scale from ‘Strongly Disagree’ to ‘Strongly Agree’. Sample items include ‘I found the system unnecessarily complex’ and ‘I felt confident using the system’. Item responses convert to a final 0–50 score, with higher values denoting better usability.

##### Cybersickness in Virtual Reality Questionnaire

Cybersickness symptoms (e.g., nausea, dizziness, eyestrain) were assessed using the CSQ‐VR (Kourtesis et al., 2023) on a 7‐point scale (1 = *No Symptoms*, 7 = *Intense Symptoms*) with a maximum of 42 points.

#### Cognitive and Psychomotor Tasks

**FIGURE 1:**
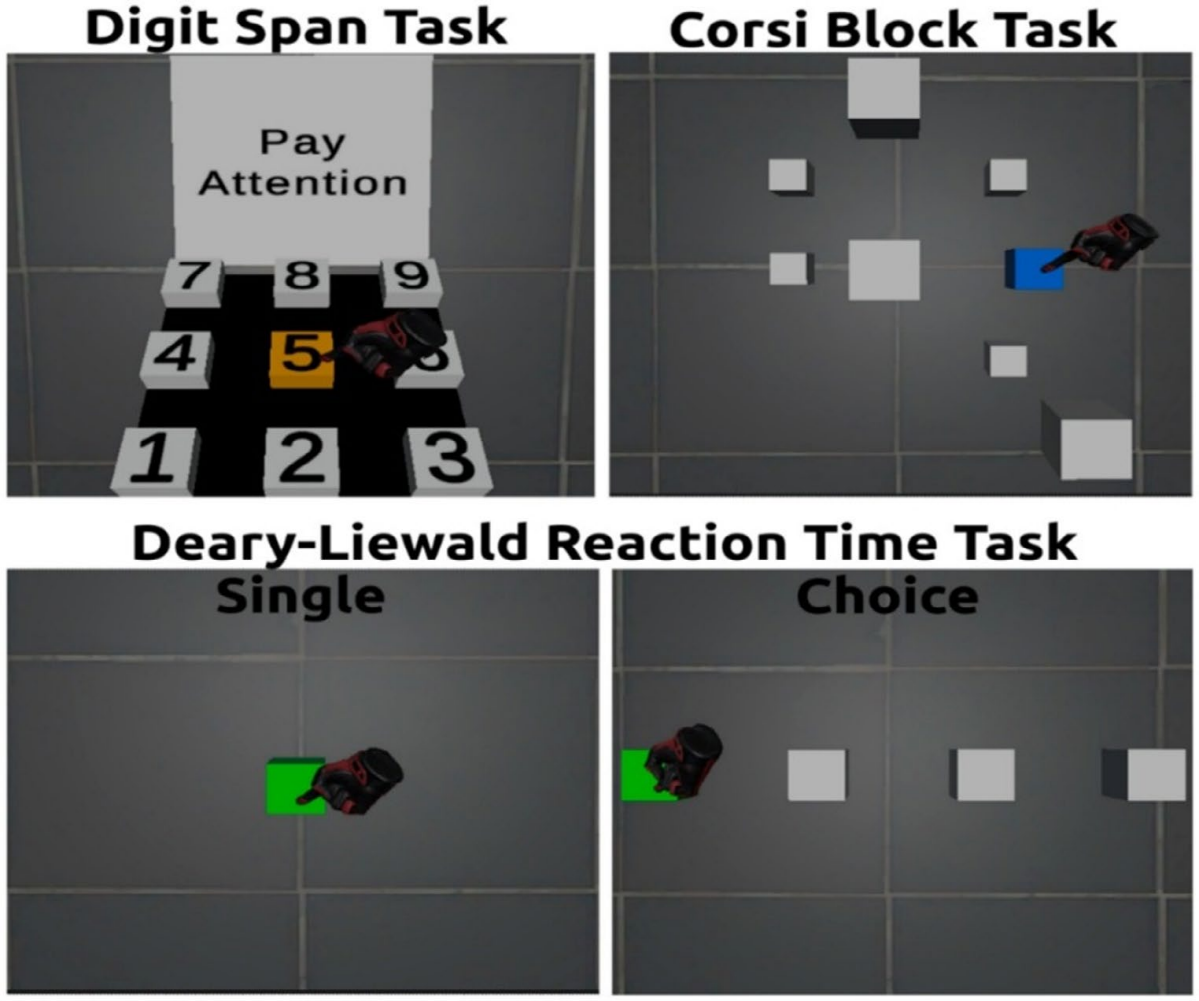
VR versions of Digit Span Task, Corsi Block Task and Deary–Liewald Reaction Time Task.

All VR implementations (Figure 1) adhered to ISO 9241‐400:2007 ergonomic requirements, adjusting object heights and reach distances to each participant's anthropometrics (International Organization for Standardization, 2007). Interaction relied exclusively on natural hand‐trajectory gestures; no buttons or keystrokes were used. A stimulus was logged as selected once the virtual hand or ray dwelled on it for ≥300 ms—an established HCI safeguard against unintentional (‘Midas‐touch’) activations (e.g., Mohan et al., 2018). Participants received neutral audio prompts and visual feedback (e.g., green for correct, orange for incorrect). A video overview of the VR versions of the tasks is available at the following links: DST, CBT, and DLRTT (accessed on 15 June 2025). The tests are compatible with any VR headset that runs on the SteamVR platform and/or uses the OpenXR framework, ensuring compatibility with the vast majority of VR headsets (Kourtesis & MacPherson, 2021). Instructions were provided in both written and auditory forms across VR and PC conditions. When absent from the PC version, spoken instructions were given by the experimenter. The substantive content was identical, while interaction‐specific elements were adapted to reflect the required input method (e.g., mouse hovering/clicking vs. controller‐based selection). Below are brief descriptions of the tasks.

##### Digit Span Task (DST)

The computerized version of the DST (Woods, Herron, et al., 2011; Woods, Kishiyama, et al., 2011) was adapted based on the original version of Ramsay and Reynolds (1995) and the version included in the Wechsler Adult Intelligence Scale (WAIS; Wechsler, 2008). Participants heard a series of digits at 2‐s intervals through headphones and then recalled forward or backward recall of the digit sequences, either by selecting digits using mouse clicks on a computerized keypad or tapping on a virtual keypad. The first sequence was of two digits’ length. At least one correct trial permitted an increase in the digit sequence length by one; two consecutive errors at the same length ended the task. The total score combined the maximum span (i.e., the maximum length with at least one trial correct at that length) and the number of correctly recalled sequences, with a maximum of 20 points for both forward and backward recall.

##### Corsi Block Task

The computerized version of CBT (Fischer, 2001) was based on the original wooden version of the Corsi (1973) Block Task to measure short‐term and working memory for spatial sequences. The CBT involves squares/cubes serially changing colour to depict the sequence order. Except for the dimensions (i.e., 2D vs. 3D objects and environment) and the response method (i.e., mouse clicks vs. virtual touching), the PC and VR versions shared comparable administration and scoring. In VR, 27 cubes were arranged in three‐dimensional space, although only nine appeared in each trial. A subset of these cubes would change colour sequentially, prompting participants to replicate the pattern either in a forward or backward order. The first sequence length was two cubes. Two sequences at the same length were presented, and the sequence length increased by one when at least one of the two sequences was correctly recalled or when the maximum span (7) was reached. The total CBT score was the sum of the highest span achieved and the number of sequences correctly recalled, with a maximum of 20 points for both forward and backward recall (see Gould & Glencross, 1990).

##### Deary–Liewald Reaction Time Task

The DLRTT was developed by Deary et al. (2011) as a computerized measure of psychomotor skills (i.e., eye‐hand co‐ordination), assessing both Simple Reaction Time (SRT) and Choice Reaction Time (CRT). In the VR‐SRT, participants had to touch a cube as quickly as possible when it changed colour from white to blue (20 trials). In the VR‐CRT, one of four cubes changed colour at random (40 trials), and participants had to touch that cube as quickly as possible. The PC version displayed similar stimuli on a monitor and required participants to press the space bar for SRT or keyboard keys (i.e., ‘z’, ‘x’, ‘,’, ‘.’) for CRT, as quickly as possible. In both versions, mean reaction times (RT) were computed. Notably, the VR version, using eye‐tracking integrated in VR headsets, is capable of measuring the time it takes for users to visually detect a target since its appearance (i.e., attentional time), as well as the interval between target detection and the selection of the target (i.e., motor time).

### Procedure

All sessions took place in a laboratory at the National and Kapodistrian University of Athens, the American College of Greece, or the University of Edinburgh. Participants completed a demographic and technological skills questionnaire, and the GSQ to indicate their familiarity with video games, smartphones, and computers. Subsequently, each participant performed three cognitive/psychomotor tasks in both VR and PC formats. Half the participants completed the VR tasks first and the other half completed the PC tasks first; within each modality, the task order was also counterbalanced. Upon completing the VR tasks, participants filled out the CSQ‐VR to assess changes in cybersickness. Finally, participants rated both modalities using the UEQ and SUS. Brief breaks were provided between tasks and modalities. The session lasted 60–90 min.

### Statistical analyses

All analyses were performed using R (version 4.1.3) (R Core Team, 2024) and the *psych* package (Revelle, 2022) in RStudio (Posit team, 2024). Each outcome variable underwent Shapiro–Wilk tests for normality, and variables that deviated significantly from normality were transformed using the *bestNormalize* package (Peterson & Cavanaugh, 2020), and then they were scaled into *z* scores to interpret the statistical analyses.

To assess convergent validity, Pearson's correlations examined relationships between the VR and PC scores for each task. Paired samples *t*‐tests were then conducted with format (VR vs. PC) as a within‐subjects factor to examine their effects on user experience, system usability and task performance. Finally, regression analyses examined whether age, education, computing experience, smartphone experience and gaming experience predicted performance on the VR and PC tasks.

Correlations among these variables were first assessed. To control for inflated type I error, *p* values from the correlation analyses were corrected for multiple comparisons using the Holm–Bonferroni method. Next, individual single‐predictor models were constructed for each variable and compared to the null model. Only those predictors that significantly improved model performance over the null were then compared against one another to identify the best‐performing variable. Building on this, additional predictors were added incrementally: in each step, the best predictor(s) from the previous stage were retained, and one new predictor was introduced. At every step, analyses of variance were used to compare models in terms of *R* ^2^ improvement, while the variance inflation factor was monitored to rule out multicollinearity.

## RESULTS

Participants reported relatively high experience of computing and smartphone applications with a mean (SD, range) of 10.21 (1.25, 4–12) and 10.46 (1.24, 6–12) respectively. Videogaming experience showed greater variability with a mean of 34.6 (12.41, 22–72). Participants reported absent to mild cybersickness symptoms in VR (see Table 1). Table 2 summarizes user experience, system usability and cognitive task performance across assessment modalities.

**TABLE 1:** Cybersickness intensity: overall and per symptom category.

	M	SD	Median	Mode	Observed range (min–max)	Possible range (min–max)
Nausea (total)	3.39	1.31	3	2	2–6	2–14
Vestibular (total)	3.28	1.27	2.5	2	2–6	2–14
Oculomotor (total)	4.05	1.64	3.5	2	2–7	2–14
Overall CSQ‐VR	10.94	4.35	9	6	6–18	6–42

*Note*: Observed ranges reflect the lowest and highest scores recorded in the present sample. Possible ranges reflect the theoretical minimum and maximum values defined by the CSQ‐VR.

**TABLE 2:** User experience, system usability and performance across format*.*

Test	Format	Mean	SD	Minimum	Maximum
UEQ (max = 56)	PC	39.27	8.91	14	55
	VR	46.12	6.79	26	56
SUS (max = 50)	PC	40.38	6.54	23	49
	VR	42.64	5.47	26	50
DSF (max = 20)	PC	16.38	2.83	8	20
	VR	16.09	3.35	0	20
DSB (max = 20)	PC	14.85	3.46	3	20
	VR	14.83	3.33	8	20
CBF (max = 20)	PC	15.79	4.39	5	20
	VR	14.85	2.64	10	20
CBB (max = 20)	PC	15.52	4.64	5	20
	VR	14.82	2.16	9	20
SRT (s)	PC	.27	.05	.20	.46
	VR	.48	.10	.27	.69
CRT (s)	PC	.42	.07	.30	.60
	VR	.56	.10	.36	.82
CRT‐AT (s)	VR	.29	.10	.18	.42
CRT‐MT (s)	VR	.23	.11	.06	.46

*Note*: AT, Attention Time; CBB, Corsi Block Task‐Backward Recall; CBF, Corsi Block Task‐Forward Recall; CRT, Deary Liewald Choice Reaction Time Task; DSB, Digit Span Task‐Backward Recall; DSF, Digit Span Task‐Forward Recall; MT, Motor Time; PC, computerized assessment; SRT, Deary Liewald Single Reaction Time Task; SUS, System Usability Scale; UEQ, User Experience Questionnaire; VR, Virtual Reality Assessment.

### Convergent validity between VR and PC versions

Across all tasks, the VR‐based measures demonstrated positive correlations with their established PC‐based counterparts, indicating convergent validity. For the Digit Span Test, VR‐based forward recall was strongly correlated with PC‐based forward recall, *r*(64) = .679, *p* < .001. The backward recall correlation was somewhat lower but still substantial, with VR‐based backward recall correlating moderately with the PC‐based version, *r*(64) = .516, *p* < .001.

The Corsi Block‐Tapping Test showed more modest but still significant correlations between the two modalities. VR‐based forward recall demonstrated a weak‐to‐moderate, yet significant, relationship with PC‐based forward recall, *r*(64) = .391, *p* = .009, while VR‐based backward recall correlated moderately with PC‐based backward recall, *r*(64) = .495, *p* < .001. Despite these lower correlations than those observed for Digit Span, they still support the convergent validity of the VR‐based Corsi tasks.

The Deary‐Liewald Reaction Time Test also showed statistically significant correlations between VR‐ and PC‐based scores. For SRT, the VR‐based measure was positively associated with the PC‐based equivalent, *r*(64) = .364, *p* = .016, indicating a modest level of convergence. The CRT measure produced a stronger correlation, with VR‐based CRT correlating robustly with the PC‐based CRT, *r*(64) = .591, *p* < .001.

Finally, inter‐task correlations within and across modalities were assessed. Both SRT and CRT did not significantly correlate with performance on either the DST or CBT tasks in either VR or PC format. Associations between the DST and CBT were consistently very weak, albeit statistically significant. Forward recall of DST and CBT demonstrated correlations, which were low within both PC, *r*(64) = .268, *p* = .042, and VR, *r*(64) = .261, *p* = .045. Comparably, backward recall of DST and CBT demonstrated weak correlations in PC, *r*(64) = .272, *p* = .037, and VR, *r*(64) = .265, *p* = .041. Cross‐task and cross‐format associations were similarly weak. VR DST Forward with PC CBT Forward, *r*(64) = .256, *p* = .048, and VR CBT Forward with PC DST Forward, *r*(64) = .264, *p* = .043.

### Comparison of formats: User experience, system usability and performance

A paired‐samples *t*‐test conducted on the UEQ showed a significant difference between the modalities, *t*(65) = −7.01, *p* < .001, Cohen's *d* = −.86, with higher ratings for VR compared to PC. The SUS scores likewise differed significantly between VR and PC, *t*(65) = −5.93, *p* < .001, *d* = −.73, again with higher usability ratings for VR. For the performance scores, there were no significant differences between the two modalities for DST forward or backward recall, *t*(65) = .63, *p* = .532, *d* = .08 and *t*(65) = −.04, *p* = .966, *d* = −.01, respectively. However, CBT forward and backward recall yielded significantly higher scores on the PC compared to the VR versions, *t*(65) = 2.46, *p* = .017, *d* = .30 and *t*(65) = 2.65, *p* = .010, *d* = .33, respectively. Reaction time tasks also showed notable differences, where faster RTs were observed in PC administration: SRTs, *t*(65) = −15.79, *p* < .001, *d* = −1.94, and CRTs, *t*(65) = −12.69, *p* < .001, *d* = −1.56.

### Predictors of the performance on VR and PC versions: Demographics and IT skills

Overall, the correlation analyses (see Table 3) revealed that participant age, computing experience and videogaming experience are substantially associated with performance on several computerized (i.e., PC‐based) tasks, while they were associated only with the VR version of CBB. In particular, age only demonstrated a significant relationship with performance on the PC version of DSB, where the older the participant, the poorer the performance, while higher videogaming experience was generally associated with better or faster performance on the PC versions of CBF, CBB and reaction times (i.e., SRT and CRT) on the DLRT. For VR tasks, correlations with demographic or IT‐experience factors were non‐significant, except for smartphone experience and videogaming experience, which were substantially and positively correlated with performance on the VR version of CBB.

**TABLE 3:** Correlations: demographics, it experience and performance on tests.

		Age	Education	Computing XP	Smartphone XP	Videogaming XP
DSF‐PC	Pearson's *r*	.18	−.02	.07	.19	.12
	*p*‐Value	.151	.877	.566	.130	.341
DSF‐VR	Pearson's *r*	.15	−.02	.15	.18	.21
	*p*‐Value	.238	.861	.074	.153	.098
CBF‐PC	Pearson's *r*	.18	−.16	.16	.10	.**24**
	*p*‐Value	.151	.189	.210	.425	.**050**
CBF‐VR	Pearson's *r*	−.01	.08	.21	.10	.10
	*p*‐Value	.976	.548	.097	.399	.424
SRT‐PC	Pearson's *r*	.11	.06	**−.24**	−.04	**−.26**
	*p*‐Value	.371	.608	.**049**	.725	.**033**
SRT‐VR	Pearson's *r*	.16	.15	−.05	−.08	−.14
	*p*‐Value	.198	.219	.683	.530	.252
CRT‐PC	Pearson's *r*	.22	.12	**−.40**	−.20	**−.36**
	*p*‐Value	.071	.339	**< .001**	.116	.**003**
CRT‐VR	Pearson's *r*	−.07	.04	−.14	−.23	−.22
	*p*‐Value	.573	.749	.261	.061	.071
CRT‐AT	Pearson's *r*	.10	−.03	.01	−.08	−.15
	*p*‐Value	.432	.823	.994	.550	.222
CRT‐MT	Pearson's *r*	.14	.07	−.02	−.20	−.23
	*p*‐value	.275	.596	.878	.108	.067
DSB‐PC	Pearson's *r*	.**31**	.12	.05	.14	.13
	*p*‐Value	.**013**	.334	.677	.260	.307
DSB‐VR	Pearson's *r*	−.11	.01	.05	.11	−.01
	*p*‐Value	.385	.939	.704	.363	.989
CBB‐PC	Pearson's *r*	.11	−.08	.**29**	.**30**	.**39**
	*p*‐Value	.366	.517	.**018**	.**015**	.**001**
CBB‐VR	Pearson's *r*	.13	.13	.18	.**32**	.**42**
	*p*‐Value	.287	.282	.155	.**009**	**< .001**

*Note*: Significant correlations are displayed in bold.

Abbreviations: AT, Attention Time; CBB, Corsi Block Task‐Backward Recall; CBF, Corsi Block Task‐Forward Recall; CRT, Deary Liewald Choice Reaction Time Task; DSB, Digit Span Task‐Backward Recall; DSF, Digit Span Task‐Forward Recall; MT, Motor Time; PC, Computerized Assessment; SRT, Deary Liewald Single Reaction Time Task; VR, Virtual Reality Assessment; XP, Experience.

These patterns were further reflected in the linear regression analyses (see Table 4). Age emerged as a significant predictor of performance on the PC versions of the DSB and CRT. However, age explained only residual variance in the CRT performance. Computing experience was a significant predictor of the PC version of the CRT. Videogaming experience was the most frequent significant predictor of performance on the PC versions of the CBF, CBB, SRT and CRT. Specifically, videogaming experience was a significant predictor of the performance on the PC versions of the CBF, CBB, SRT and CRT. Notably, the best model explained 27% of the variance on the PC version of the CRT. By contrast, the null models were best for explaining performance on the VR versions of the tests, which postulates that demographics and/or IT experience do not substantially modulate performance on the VR versions. Comparably to the PC version, videogaming experience was found to predict performance on the VR‐based CBB.

**TABLE 4:** Best linear regression models: demographics and IT skills as predictors of performance on cognitive tests/tasks.

Predicted	Predictors	*β* coefficient	*p*‐Value (*β*)	*R* ^2^
DSF‐VR	Null Model	–	–	–
DSF‐PC	Null Model	–	–	–
DSB‐VR	Null Model	–	–	–
DSB‐PC	Age	.30	.013	.10
CBF‐VR	Null Model	–	–	–
CBF‐PC	Videogaming XP	.17	.033	.07
CBB‐VR	Videogaming XP	.25	<.001	.17
CBB‐PC	Videogaming XP	.49	<.001	.15
SRT‐VR	Null Model	–	–	–
SRT‐PC	Videogaming XP	‐.17	.033	.07
CRT‐VR	Null Model	–	–	–
CRT‐AT	Null Model	–	–	–
CRT‐MT	Null Model	–	–	–
CRT‐PC	Age	.14	.018	.27
	Videogaming XP	−.14	.037	
	Computing XP	−.15	.026	

Abbreviations: AT, Attention Time; CBB, Corsi Block Task‐Backward Recall; CBF, Corsi Block Task‐Forward Recall; DSB, Digit Span Task‐Backward Recall; DSF, Digit Span Task‐Forward Recall; MT, Motor Time; PC, Computerized Assessment; SRT, Deary Liewald Single Reaction Time Task; VR, Virtual Reality Assessment; XP, Experience.

## DISCUSSION

The present study examined the usability, convergent validity and performance differences between VR‐based and traditional PC‐based neuropsychological assessments of working memory and psychomotor skills. Our findings show that VR assessments achieve convergent validity with PC‐based tests while offering enhanced engagement with assessments that better resemble the complexity and cognitive demands of the real world. Importantly, regression analyses revealed that VR performance was less influenced by demographic factors and IT‐related skills—such as computing and gaming experience—than PC‐based tasks. These results suggest that VR can provide a more inclusive, realistic and less‐biased platform for neuropsychological evaluation.

### Validity, user experience and usability of VR adaptations of traditional tasks

The introduction of 3D spatial components and naturalistic interactions in VR tasks may invoke cognitive processes that more accurately mirror everyday problem‐solving, navigation and decision‐making (Kourtesis & MacPherson, 2021; Parsons, 2015). This could explain the performance differences between the VR and PC versions, where faster reaction times on the DLRTT and better visuospatial memory on the CBT were observed. For example, the VR version of the CBT required encoding and recalling spatial sequences along the *z*‐axis, adding depth perception that engages real‐world‐like navigation processes more than the 2D PC version. Consistent with this, recent evidence shows that learning and memory outcomes differ systematically across VR, 3D desktop, and 2D desktop modalities, with immersive VR producing distinct encoding advantages (Barrett et al., 2022). Moreover, immersive VR imposes higher cognitive load than 2D formats, as shown by greater frontal‐midline theta activation during the encoding phase of visuospatial tasks (Slobounov et al., 2015), indicating that additional attentional control processes are recruited. Similarly, embodied interactions for target selection, albeit intuitive and ergonomic, required longer movement trajectories compared to key pressing (Kourtesis et al., 2022). Hence, VR adaptations of the tests impose extra spatial and motor demands to better mirror real‐world interactions. As expected, the DST showed negligible VR–PC differences, consistent with its role as a verbal working‐memory control task and validating our design rationale to contrast it with the more visuospatially demanding CBT and DLRTT.

Importantly, although several VR–PC correlations were below .70, they nonetheless fall within the moderate range considered acceptable. Previous validation studies confirm this pattern, where immersive VR assessments consistently correspond with established neuropsychological measures (Climent et al., 2021; Kourtesis et al., 2021); tablet‐based and traditional Corsi tasks correlate moderately (Siddi et al., 2020); and VR reaction‐time tasks converge with computerized versions (Loushy Kay et al., 2025). Meta‐analyses of VR neurocognitive tools further support this interpretation, reporting pooled medium correlations around *r* = .51 with conventional (computerized and paper‐and‐pencil) neuropsychological tests, with observed values ranging between *r* = .35 and *r* = .70, and the majority clustering in the .40–.60 range (Lee et al., 2024; Negu et al., 2015). Hence, the correlations observed in this study fall squarely within the expected range and substantially support the convergent validity of the VR adaptations of the examined tests.

Moreover, beyond the demonstrated convergence between PC versions and VR adaptations of the tests, the very weak but statistically significant associations between the DST and CBT tasks, both within and across modalities (VR and PC), further support the interpretation that these tasks assess distinct components of working memory (i.e., verbal and visuospatial respectively). Similarly, the absence of significant correlations between the DLRTT and the memory tasks reinforces its role as a domain‐specific measure of psychomotor speed and attentional processing (Deary et al., 2011). Therefore, the current findings not only validate the construct‐specificity of these well‐established cognitive measures, but also confirm that their VR adaptations preserve these distinctions. This supports the use of immersive VR as a viable and psychometrically sound platform for assessing multiple cognitive domains without artificially inflating inter‐task correlations due to shared modality artifacts or interface requirements.

Moreover, cybersickness symptoms were uniformly low across participants and never exceeded mild levels on any subscale, suggesting that immersive VR was well tolerated in our young adult sample. This tolerability complements the broader usability and user experience findings, where VR assessments received significantly higher ratings than PC methods. Consistent with prior studies (Kourtesis & MacPherson, 2021), our VR assessments received significantly higher user experience and usability ratings than PC methods, suggesting that immersive VR substantially enhances participant engagement with cognitive tasks, as well as inclusivity. Higher user experience likely stems from the immersive nature of VR (Mäkinen et al., 2022; Slater, 2018), as well as the emulation of real‐world dimensions and challenges (Slater, 2009; Slater & Sanchez‐Vives, 2016); the assessments also resemble better the complexity and cognitive demands of everyday tasks (i.e., verisimilitude) (Kourtesis & MacPherson, 2021).

Furthermore, user experience is also modulated by the usability of the system (Kim et al., 2020; Mäkinen et al., 2022), especially in clinical populations (Tuena et al., 2020) and older adults (Abeele et al., 2021; Shao & Lee, 2020). Participants rated the VR versions of the assessments as highly usable, which may further explain the comparably high user experience ratings. Given that the VR versions were developed to offer ergonomic (Kourtesis et al., 2022) and naturalistic (Kourtesis et al., 2020) interactions, these findings are in line with existing literature reviews with healthy young (Kim et al., 2020) and older adults (Abeele et al., 2021), and clinical populations (e.g., Parkinson's or Alzheimer's disease; Tuena et al., 2020). Notably, in line with the current study's findings, the evidence‐based guidelines for neuropsychological testing (Kourtesis et al., 2020) and psychological applications in older adults (Abeele et al., 2021) highlight the importance of intuitive interactions and how these may enhance user experience, usability, and acceptability of the respective VR software.

A move from mouse clicks in PC tasks to hand‐tracked interactions in VR appears to reduce the influence of fine motor skills and digital familiarity, potentially mitigating performance disparities linked to technological competency. This reduced dependency on specific motor skills suggests that VR assessments may be more accessible and fairer, especially for populations facing barriers with traditional PC testing. Lastly, heightened user experience in VR tasks may reduce fatigue and boredom, leading to more reliable data in lengthy assessments or with fatigue‐prone populations like older adults or those with cognitive impairments (González‐Erena et al., 2025; Tuena et al., 2020).

### Effects of demographics and IT skills on performance

Regression analyses highlighted that demographic factors and IT‐related skills, such as age, computing, and gaming experience, significantly influenced PC‐based task performance, whereas their impact on VR assessments was minimal. For instance, while videogaming experience modestly predicted performance on the VR version of the CBT backward recall, this relationship was weaker than expected and absent for VR forward recall—contrasting with the PC version where the corresponding relationships were significantly strong. Also, while gaming and computing experience accounted for a considerable amount of variance on PC‐based reaction time tasks, these factors did not significantly affect performance on VR‐based equivalents, suggesting that intuitive VR interactions mitigate advantages from prior digital literacy. These findings are in line with the relevant studies on traditional computerized assessments (see Feldstein et al., 1999; Iverson et al., 2009), where IT experience was found to affect performance, as well as the studies on motor tasks in VR (seeKourtesis et al., 2022; Zaidi et al., 2018) where gaming experience did not influence performance.

The effects of digital proficiency on DLRTT performance evidently demonstrated how prior experience can bias test outcomes. In the PC version, videogaming experience significantly predicted both SRT and CRT, and when combined with general computer use and age, it explained up to 27% of the variance in CRT. However, age by itself showed no significant link to either reaction‐time measure (CRT or SRT) in any format (PC or VR), indicating that its role in the PC CRT model does not reflect genuine cognitive ageing. Age became a predictor only after the variance attributable to gaming and computing was removed, suggesting it merely captures residual familiarity with mouse‐and‐keyboard interaction. Also, because the sample consisted of young adults, true age‐related decline is improbable, further reinforcing the interpretation of a sham age effect. Consistently, no age effects appeared in any VR task, whether in simple correlations or regression analyses. Overall, immersive VR eliminates these spurious ‘age’ signals—actually artefacts of the interaction modality—and offers a fairer, cognition‐focused platform for assessing psychomotor and attentional performance across participants of varied ages and digital backgrounds.

Finally, a key advancement in the VR version of DLRTT was the incorporation of eye‐tracking, which allowed for the separation of attentional time (detection of visual stimulus) from motor time (execution of motor response) in CRT. Notably, neither attentional nor motor time was found to be affected by prior IT or gaming experience of the examinee. This distinction is critical for isolating the specific components of reaction time, a feature that traditional PC‐based assessments lack. By independently measuring these components, eye‐tracking enhances the precision of psychomotor assessments and provides deeper insights into where specific delays or inefficiencies may occur. Given that the DLRTT is a tool that can be used in cognitive ageing (Deary & Der, 2005a; Der & Deary, 2006), which can predict onset of age‐related disorders and mortality (Deary & Der, 2005b; Der & Deary, 2006), the advancements introduced in the VR version should be further examined in the context of cognitive ageing and/or neurodegeneration.

### Implications for neuropsychological assessment methodologies

The findings of this study have broader implications for neuropsychological testing, as well as researchers examining the effects of videogaming or general IT skills on cognitive performance. Specifically, our findings postulate that traditional computerized tasks confound cognitive assessment with technological proficiency, which aligns with previous studies (e.g., Feldstein et al., 1999; Iverson et al., 2009) and the joint position of the American Academy of Clinical Neuropsychology (AACN) and the National Academy of Neuropsychology (NAN) (Bauer et al., 2012). Hence, the use of traditional computerized tasks may conflate the effects of videogaming or general IT skills on cognitive performance due to enhanced fine motor skills. Adopting testing formats like VR or paper‐and‐pencil assessments, which minimize such confounds, may be advantageous. In line with previous work examining intuitive interaction within immersive VR, which allows non‐gamers to perform standard cognitive tasks comparably to gamers (Kourtesis et al., 2022; Zaidi et al., 2018), this study's findings postulate that VR‐based tasks do not necessitate the same level of fine motor coordination or familiarity with digital interfaces, thus providing a purer assessment of the cognitive constructs under investigation.

Furthermore, by reducing biases associated with demographic factors and IT proficiency, VR assessments can create more inclusive environments for diverse populations. This meets point 6 (*Examinee issues: Cultural, experiential and disability factors*) of the criteria of the AACN and NAN (Bauer et al., 2012). The enhanced engagement reported in immersive VR tasks also suggests reduced fatigue and boredom during testing, crucial for obtaining accurate cognitive assessments, especially in clinical settings involving children, older adults or patients with cognitive impairments (Borghetti et al., 2023; Chiossi et al., 2025; González‐Erena et al., 2025).

### Limitations and future directions

One limitation of this study is that the PC comparison used mouse‐based interaction rather than a touch‐screen interface, which is increasingly common in clinics and may yield different usability outcomes. While the examined modalities (desktop/laptop with mouse and/or keyboard input) are widely used, future work should extend validation to tablet‐based versions and compare them in terms of usability and user experience. Moreover, while usability and user experience were measured with validated instruments (SUS, UEQ), these tools do not assess hardware‐specific or environmental issues (e.g., headset weight, heat, sweating). Future research should complement standardized scales with targeted measures of headset tolerability, particularly in older or clinical populations.

Furthermore, while this study provided robust evidence that VR tasks mitigate the confounding effects of IT skills on performance, our predominantly young, tech‐savvy sample limits the generalizability of findings to older adults or less technologically familiar populations, where the effects are expected to be more pronounced. Moreover, our findings on cybersickness and usability may not generalize to older adults or clinical groups. Even if young participants tolerated VR well, other populations might face greater headset discomfort, fatigue, or resistance to immersive formats. Future studies should therefore evaluate long‐term tolerability and acceptance across more diverse cohorts, especially the examination of IT skills effects on hand‐eye coordination tasks (e.g., DLRTT) in older adults should be prioritized. In addition, integrating multisensory inputs (e.g., auditory, haptic) and examining how VR's spatial and motor requirements affect different populations could further refine task design. Longitudinal studies are also needed to assess the reliability and sensitivity of VR‐based assessments over time, especially for detecting early cognitive decline or monitoring neurological conditions. Evaluating VR's utility in clinical populations (e.g., neurodegenerative diseases) could pave the way for targeted, adaptive immersive VR‐based interventions, thereby enhancing both diagnostic precision and therapeutic outcomes.

## CONCLUSION

This study highlights the potential of immersive VR‐based assessments as engaging alternatives compared to traditional computerized neuropsychological tests. By introducing three‐dimensional spatial components, naturalistic interactions, and eye‐tracking metrics, VR assessments extend and enrich computerized methods. Our findings indicate that VR‐based tools not only replicate established cognitive measures but also minimize biases associated with prior IT proficiency, offering a more inclusive and realistic evaluation of cognitive and motor functions. As VR technology advances, it may pave the way for the next generation of automated cognitive testing and intervention tools, offering comprehensive, accurate and highly engaging platforms that closely mirror the complexities of real‐world cognitive function.

## AUTHOR CONTRIBUTIONS

**Panagiotis Kourtesis:** Conceptualization; methodology; software; formal analysis; visualization; writing – original draft; writing – review and editing; project administration; supervision. **Andrea Lizarraga:** Investigation; data curation; writing – review and editing; writing – original draft. **Sarah E. MacPherson:** Conceptualization; methodology; writing – review and editing; supervision; project administration.

## FUNDING INFORMATION

The study received no external funding.

## CONFLICT OF INTEREST STATEMENT

The authors declare no conflicts of interest.

## Data Availability

The data that support the findings of this study are available from the corresponding author upon reasonable request. The data sets are not publicly available due to restrictions in the study's ethical approval, which did not permit deposition in a public repository.
